# Supplementary material for: Dedicated surveillance mechanism controls G-quadruplex forming non-coding RNAs in human mitochondria
Source: Nat Commun. 2018 Jul 2;9:2558. doi: 10.1038/s41467-018-05007-9 (PMC6028389; doi:10.1038/s41467-018-05007-9)
Supplement: Supplementary file 1 — Supplementary Information [file 41467_2018_5007_MOESM1_ESM.pdf]

SUPPLEMENATRY FIGURES AND TABLES

Dedicated surveillance mechanism controls G-quadruplex forming non-coding RNAs in human mitochondria

Pietras et al.

SUPPLEMENTARY FIGURES

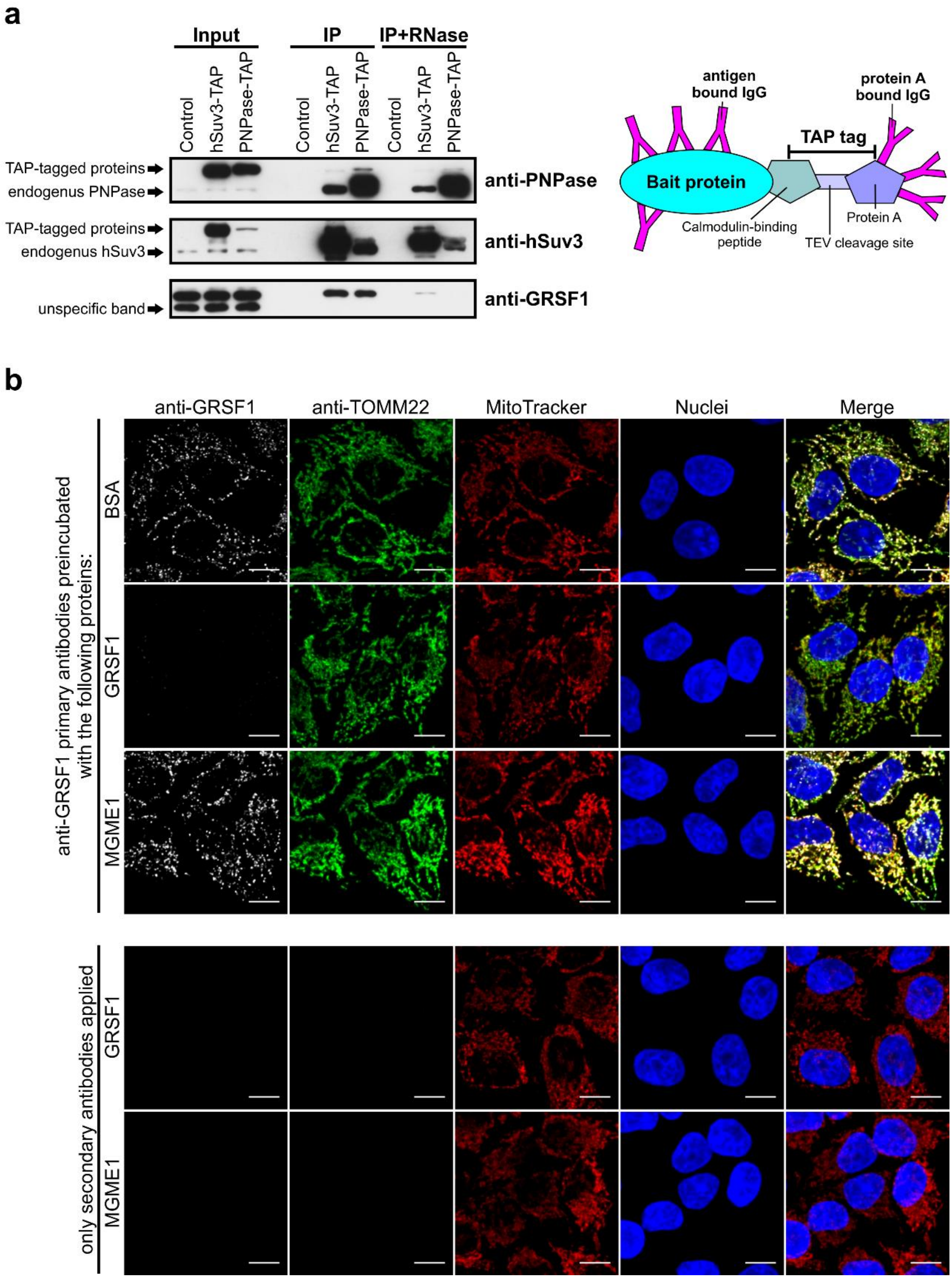

Supplementary Figure 1. GRSF1 associates with mitochondrial degradosome components in RNA dependent manner. **a** Western analysis of IP fractions from hSuv3 and PNPase purifications, as control TAP-tag targeted to mitochondria was used. Protein extracts were divided into two equal fractions and one of them was treated with RNase A (20 μg/ml) for 15 minutes at 4°C before mixing with affinity beads. Please note that TAP-tagged proteins are detected in the input fractions because the heavy chain of antibodies within the Fc region binds protein A (TAP-tag component) – schematic sketch on the right. **b** Specificity tests of anti-GRSF1 antibodies used for immunofluorescence detection of GRSF1 (related to main Fig. 1b). Prior to the immunofluorescence staining anti-GRSF1 antibodies were incubated with indicated proteins. Mitochondria were stained with anti-TOMM22 antibodies and with Mitotracker DeepRed Dye. Nuclei were stained with Hoechst 33342 dye. Scale bars represent 10 μm.

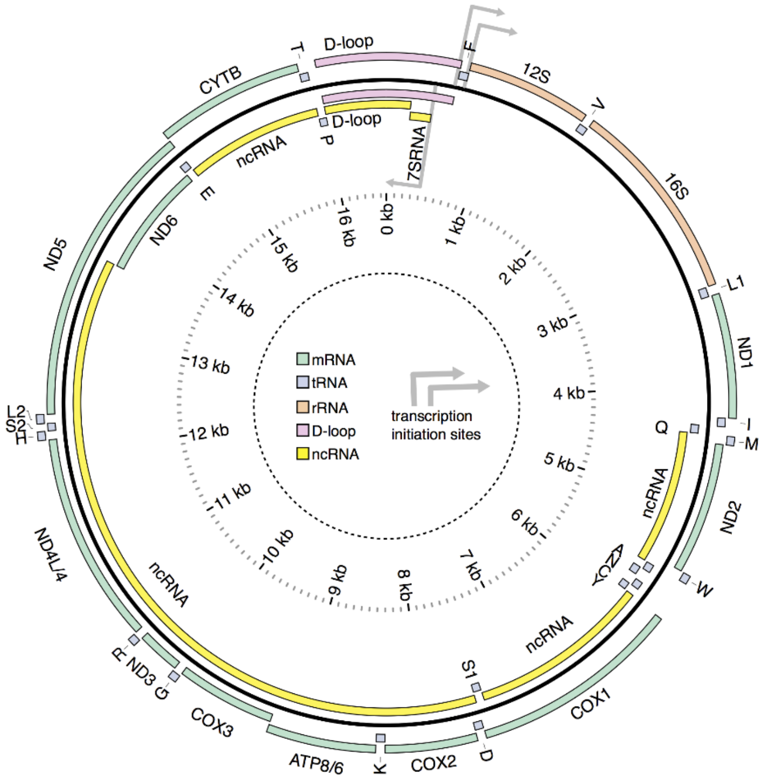

Supplementary Figure 2. Map of transcripts encoded by the human mitochondrial genome. Transcripts arising from the transcription of H-strand and L-strand of mtDNA are marked as outer and inner tracks, respectively.

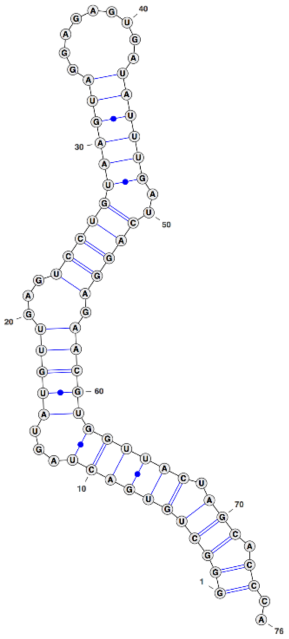

Supplementary Figure 3. Predicted secondary structure of tRNA-like. Prediction was done using the RNAfold web server (http://rna.tbi.univie.ac.at/cgi-bin/RNAWebSuite/RNAfold.cgi).

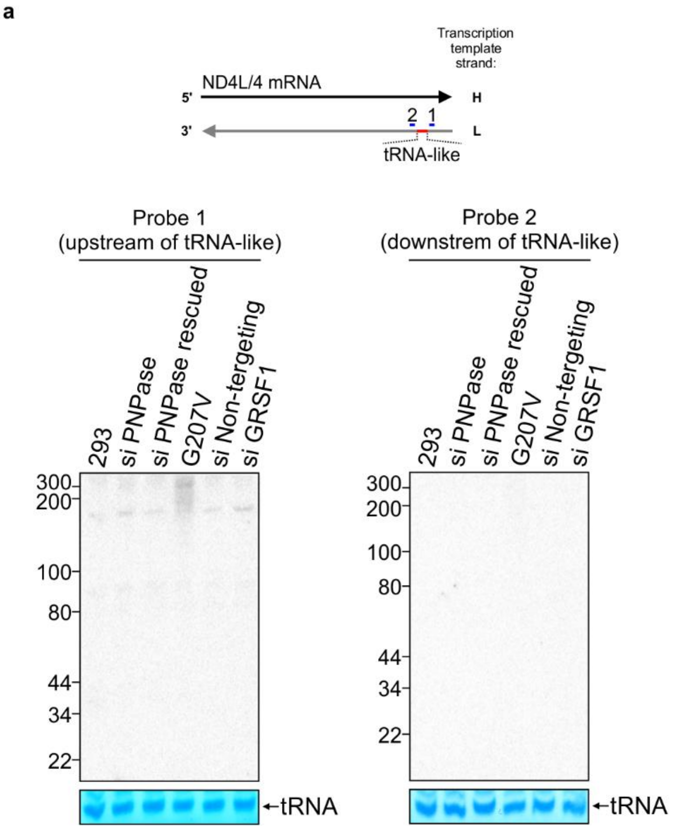

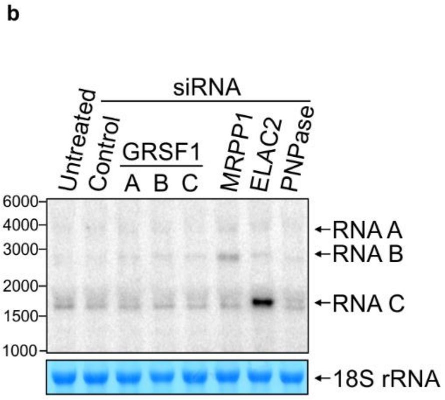

Probe	Name	Sequences that may be contained within detected RNA species	Estimated size of detected RNA
tRNA Val	RNA A	(Phe)+12S+Val+16S+Leu+ND1+(Ile)+(Met) = **3615 (3892)**	3.4
	RNA B	(Phe)+12S+Val+16S+(Leu) = **2581 (2727)**<br>or<br>Val+16S+Leu+ND1+(Ile)+(Met) = **2656 (2867)**	2.8
	RNA C	Val+16S+(Leu) = **1627 (1702)**	1.7

Supplementary Figure 4. Confirmation of tRNA-like encoding region and efficient silencing of enzymes involved in processing primary polycistronic mitochondrial transcripts. **a** The same RNA sample as that analyzed for Figure 3a-e presented in the main text was analyzed by northern blot. A diagram of applied strand-specific oligonucleotide probes is shown at the top. Methylene blue staining of tRNA is shown as a loading control. A membrane presented on the left panel was also used for hybridization presented on Figure 3e in the main text. **b** The same RNA as that analyzed for Figure 3f was analyzed by northern blot. A strand-specific oligonucleotide probe detecting tRNA Val was used. Unprocessed precursors are indicated with arrows. We detected the same precursors as previously reported by Brzezniak et al.[1], who described the involvement of ELAC2 in tRNA excision from polycistronic transcripts and its interplay with MRPP1. Because the analysis resolution did not allow the unambiguous assignment of mtRNAs to detected precursors, a table presenting possible combinations is shown below the autoradiogram.

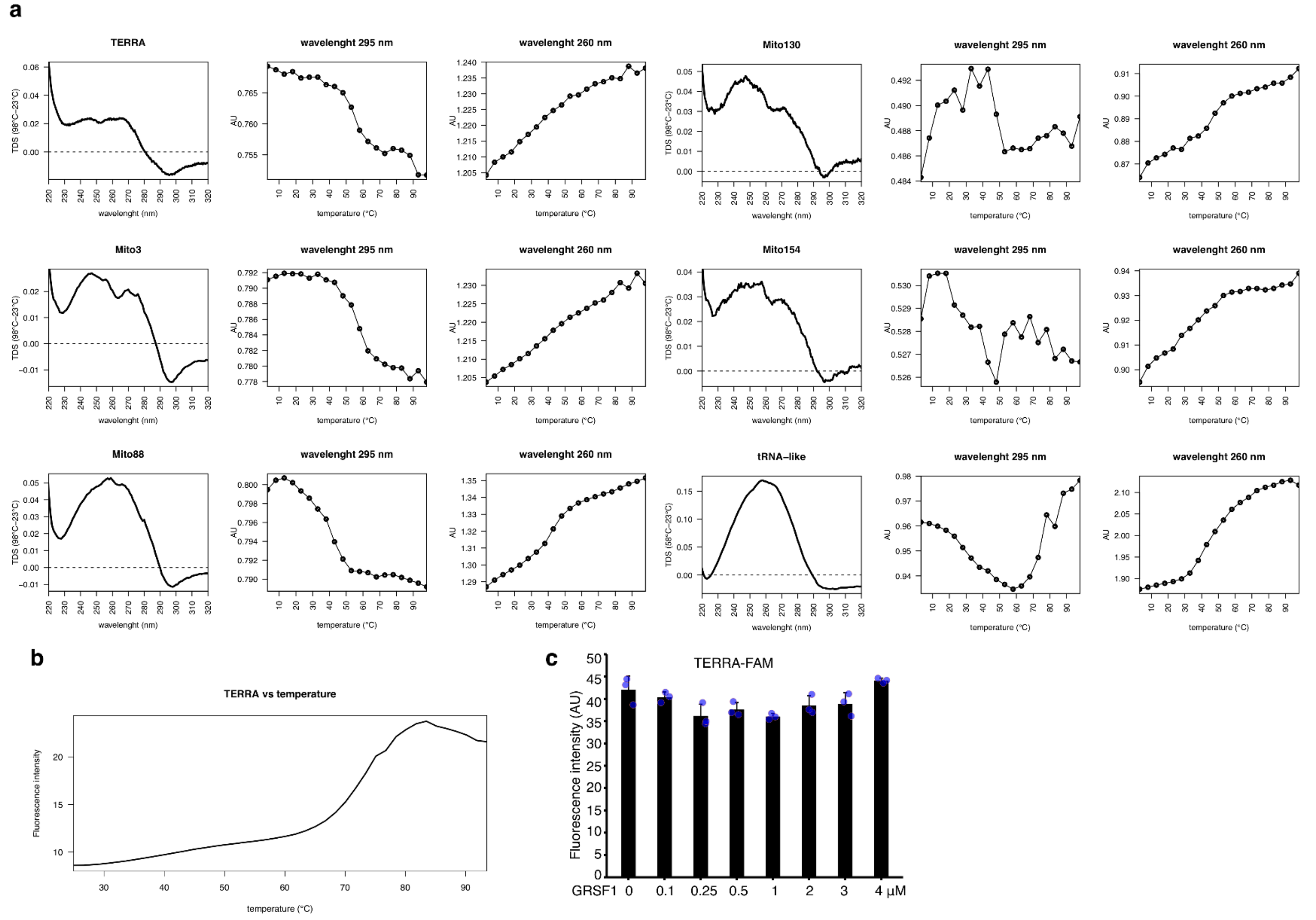

Supplementary Figure 5. Thermal denaturation studies of RNA oligomers. **a** From left: TDS and absorbance measured during thermal denaturation of the indicated RNA oligos. **b** Temperature response of fluorescence signal for TERRA RNA oligomer labeled with fluorophore and quencher. **c** GRSF1 does not change fluorescence intensity of TERRA RNA labeled with just fluorophore. Bars represent average values from three independent replicates. Error bars represent standard deviation. Blue dots represent individual values.

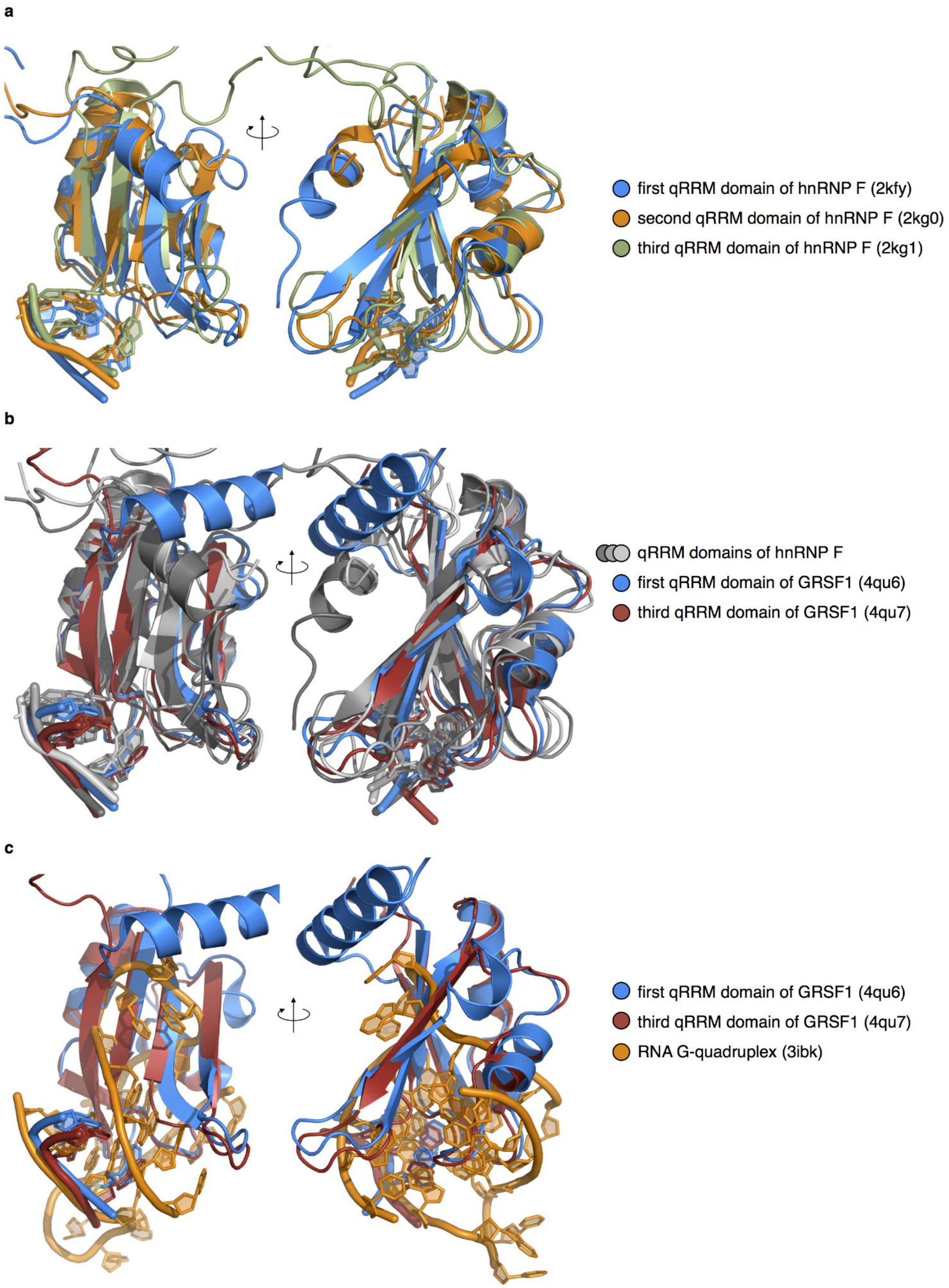

Supplementary Figure 6. Structural analysis of RNA binding by GRSF1. **a** Superposition of three qRRM domains of hnRNP F. Bound RNA is positioned at the bottom. **b** Superposition of qRRM domains of hnRNP F with two qRRM domains of GRSF1 with bound RNA. **c** qRRM domains of GRSF1 with superpositioned G4 RNA shows multiple spatial clashes.

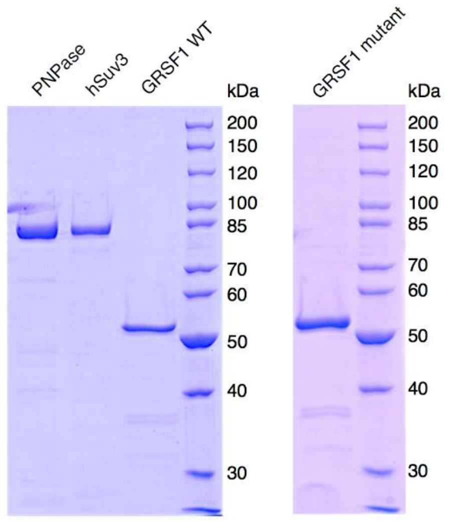

Supplementary Figure 7. Electrophoretic analysis of purified recombinant proteins. The indicated purified proteins were subjected to SDS-PAGE. After electrophoresis, the proteins were stained with Coomassie Brilliant Blue.

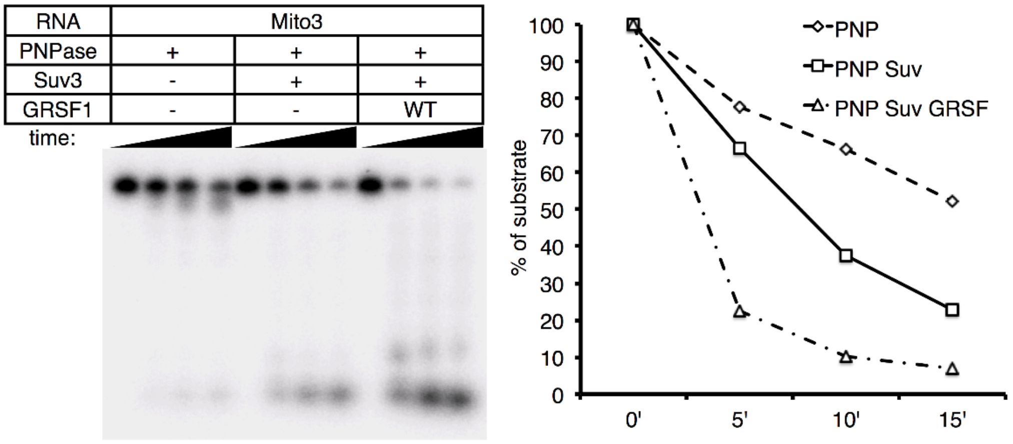

Supplementary Figure 8. GRSF1 augments degradosome-mediated degradation of G4 RNA. *In vitro* degradation of Mito3 substrate by recombinant PNPase, hSuv3, GRSF1 or their combined action. Graphs show quantified substrate decay. Following time points were analysed: 0, 5, 10 and 15 minutes. Data points represent quantification of single experiment.

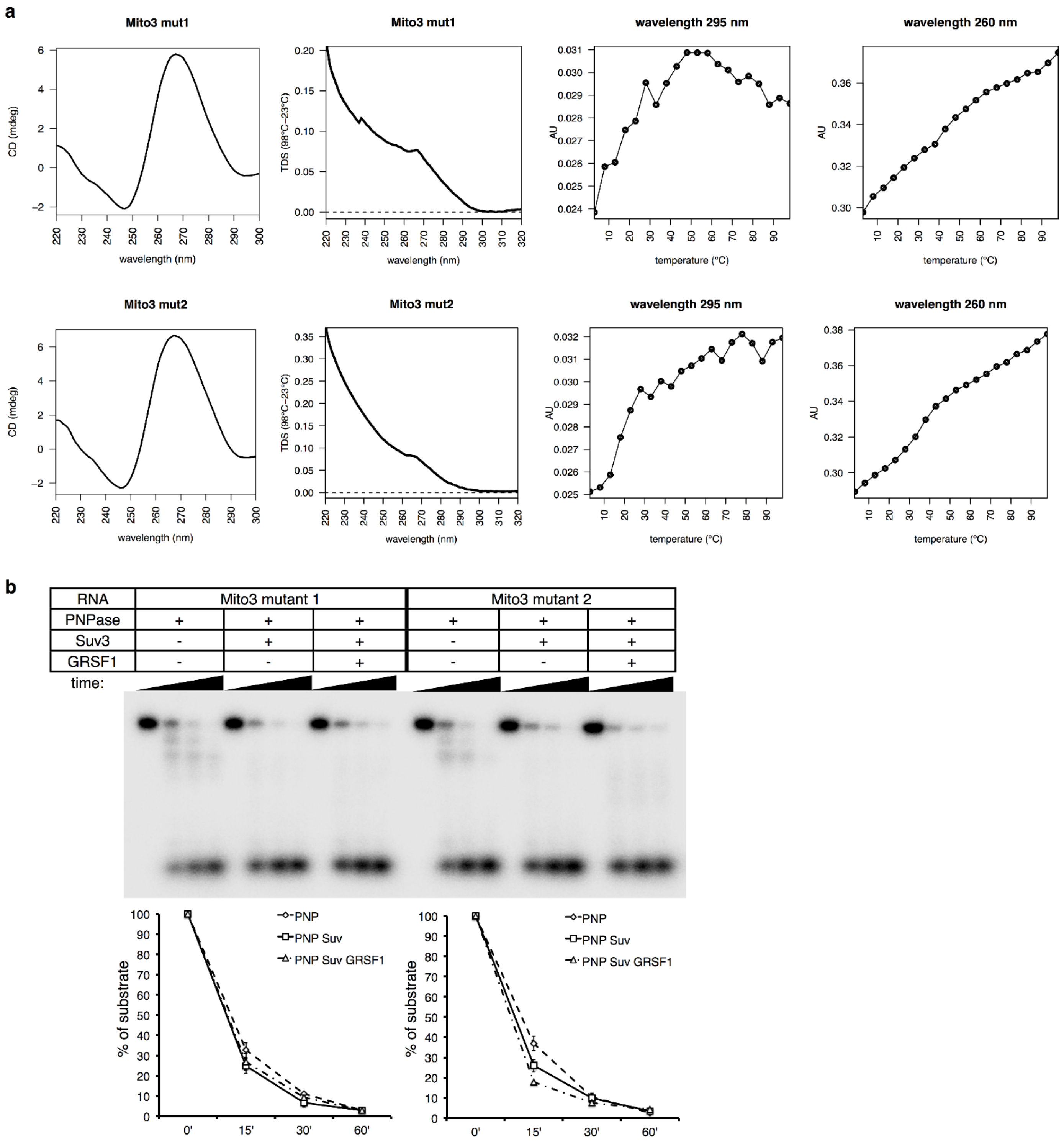

Supplementary Figure 9. Mutation of G-tracts abolishes G4 formation and GRSF1 dependent degradation. **a** From left: CD spectra, TDS and absorbance measured during thermal denaturation of the indicated RNA oligos. **b** *In vitro* degradation of indicated substrates by recombinant PNPase, hSuv3, GRSF1 or their combined action. Graphs show quantified substrate decay. Following time points were analysed: 0, 15, 30 and 60 minutes. Data points represent the mean for three independent experiments. Error bars represent standard deviation.

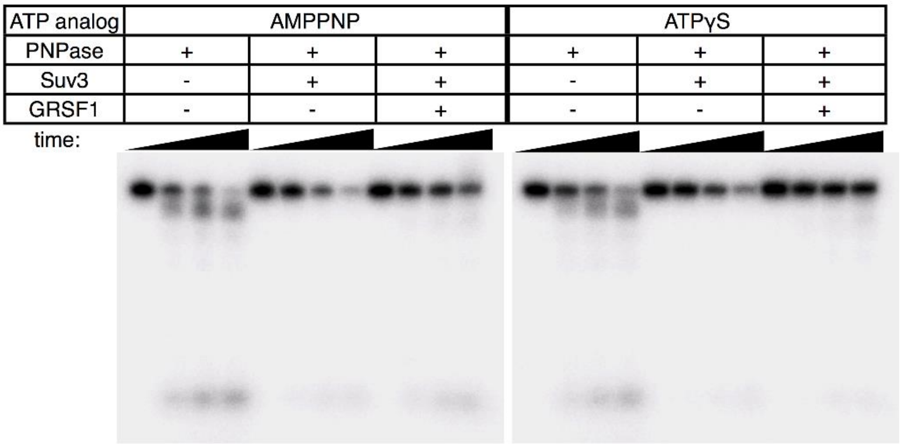

Supplementary Figure 10. G4 RNA degradation in the presence of non-hydrolysable ATP analogs. *In vitro* degradation of Mito3 substrate with indicated non-hydrolysable ATP analogs by recombinant PNPase, hSuv3, GRSF1 or their combined action. Following time points were analysed: 0, 15, 30 and 60 minutes.

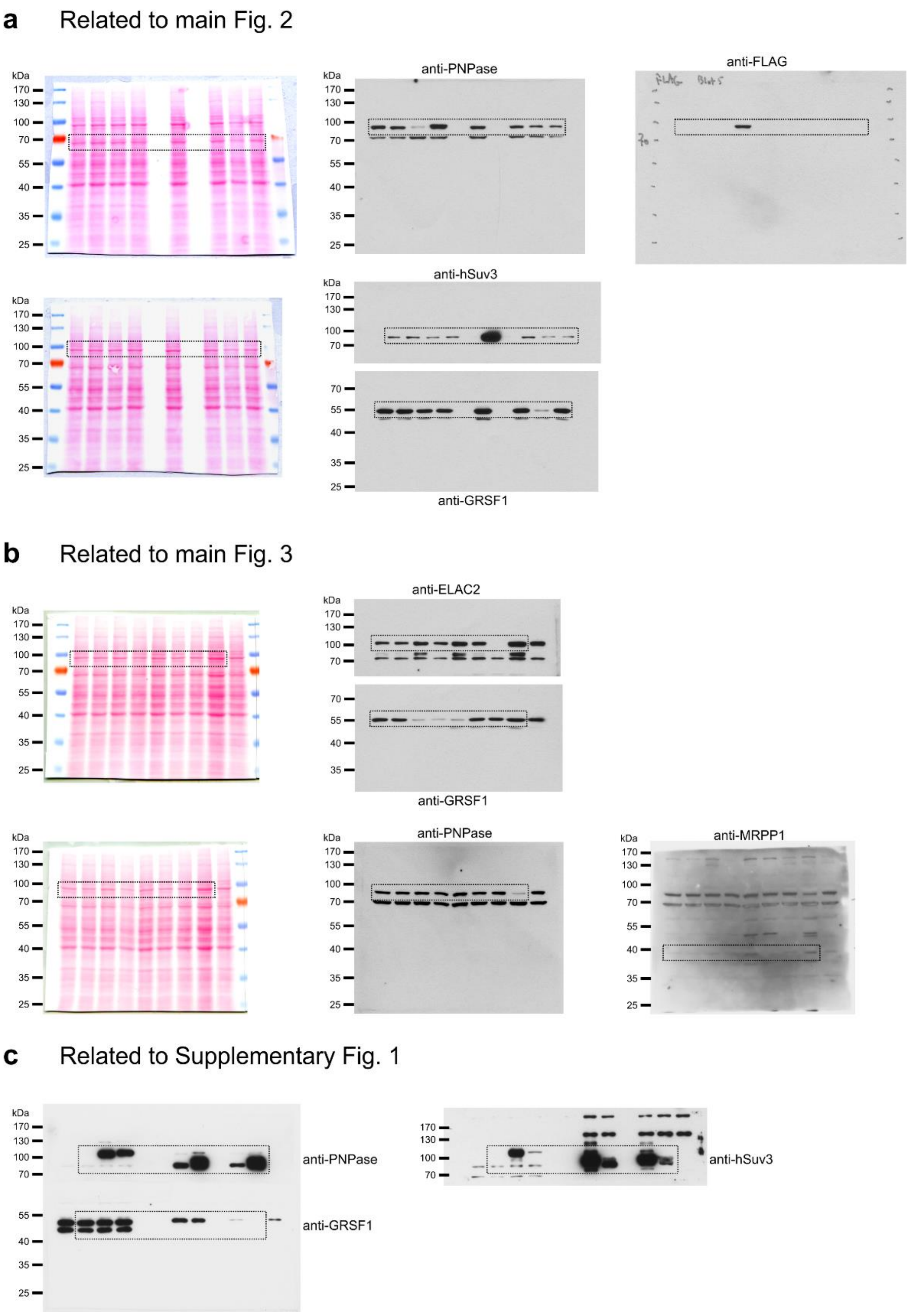

Supplementary Figure 11. Uncropped images of western blots. **a** Ponceau S staining and western blots related to Fig. 2e. **b** Ponceau S staining and western blots related to Fig. 3g. **c** Western blots related to Supplementary Fig. 1.

SUPPLEMENTARY TABLES

Supplementary Table 1. Oligoribonucleotides used in the study. Synthetized by FutureSynthesis (Poznan, Poland)

Name	Sequence	Reference
Mito3	GAAGCGGGGGAGGGGGGGUUUGGUGGAAAUUUU	2, modified*
Mito3 mut1	GAAGCGGAGGAGAGCGCGUUUGAUGAAAAUUUU	This study
Mito3 mut2	GAAACGGAGGAGGGCGCGUUUGAUGAAAAUUUU	This study
Mito4	GGUGUUAGGGUUCUUUGUUUUUGGGGUUUGGCAGAGA	2
Mito88	AAUUGGGGGGUAGGGGCUAGGCUGGAGUGGUAAAAGGCUCAGAA	2
Mito130	GGCGUUUAAUGGGGUUUAGUAGGGUGGGGUUAUUU	2
Mito154	GAGUGUGGGUUUAGUAAUGGGGUUUGUGGGGUUUUCU	2
tRNA-like	GGGCUGUGACUAGUAUGUUGAGUCCUGUAAGUAGGAGAGUGAUAUUUGAUCAGGAGAACGUGGUUACUAGCACCCA	This study
tRNA-likeMutant	GCGCUGUGACUAGUAUGUUGAGUCCUGUAAGUAGCAGAGUGAUAUUUGAUCACGAGAACGUCGUUACUAGCACCCA	This study
TERRA	UUAGGGUUAGGGUUAGGGUUAGGGAAAAAAAAAA	3, modified*
TERRA-quench	Fluorescein-UUGGGUUAGGGUUAGGGUUAGGGUU-dabcyl	4
Use1	GUAGCAGGGCGGACCUCGGAGGGGAAGGACCUCACUCAAAAAA	5, modified*
NRAS	AGGGAGGGGCGGGUCGGGAAAAAAAAAA	3, modified*
ssRNA	CAGUGUCCUCGUGCUCCAGUCG	This study
dsRNA oligo 1	CAGUGUCCUCGUGCUCCAGUCG	This study
dsRNA oligo 2	CGACUGGAGCACGAGGACACUGACAUGGACUGAAGGAGUAGAAA	6
GNRA	GGGCGGGCGCAAGCCCGCCCAUUAUUA	7, modified*

* – sequence of oligonucleotide was changed and differs from the one present in the indicated reference; added nucleotides are underlined.

Supplementary Table 2. Analysis of mitochondrial genomes of GRSF1-positive and -negative organisms. A total of 10 GRSF1-positive and 3 GRSF1-negative (lancelet, fly, worm) organisms were analyzed. GC skew, %AT and genome length are shown. QGRS Mapper[8] was used to identify G4 sequences. Two numbers of identified G4 sequences are shown: 1) overlapping of G4 sequences is not allowed, 2) overlapping of G4 sequences is possible (a given G-tract can be involved in forming more than one G4 structure). Additionally, the number of G4 sequences was calculated using regular expression (Regex) (4 stretches of at least 3 Gs spaced by up to 30 nucleotides). The same regular expression was used for identification of G4 sequences in 10,000 artificially generated sequences of the same length, AT content and GC skew as the actual mitochondrial genome of each tested species. Average number of G4 consensuses within those artificially generated sequences is shown.

GRSF1	Species	GCskew	%AT	Length (bp)	QGRS Mapper		Regex (3G,30N,3G)	Average G4 content in 10,000 generated genomes (3G,30N,3G)	Times found	One tailed p-value
					no overlap	with overlap				
√	Homo sapiens (human)	-0.41	56%	16569	176	7203	35	21.06	9	0,0009
√	Macaca mulatta (rhesus)	-0.41	57%	16564	149	4542	26	18.08	313	0,0313
√	Mus musculus (mouse)	-0.33	63%	16299	86	1330	4	3.48	4650	0,4650
√	Gallus gallus (bird)	-0.41	54%	16775	201	9359	42	28.27	24	0,0024
√	Anolis carolinensis (reptile)	-0.27	62%	17223	100	1761	11	3.1	4	0,0004
√	Xenopus laevis (amphibian)	-0.28	63%	17553	77	759	6	2.67	514	0,0514
√	Danio rerio (fish)	-0.20	60%	16596	88	879	6	2.9	706	0,0706
√	Callorhinchus milii	-0.24	66%	16769	54	539	2	0.86	2148	0,2148
√	Latimeria chalumnae	-0.28	58%	16407	114	1367	12	7.39	636	0,0636
√	Lepisosteus oculatus	-0.28	58%	16330	134	2550	12	7.39	680	0,0680
X	Branchiostoma floridae (lancelet)	0.18	65%	15083	56	410	3	0.65	281	0,0281
X	Drosophila melanogaster (fly)	-0.17	82%	19517	8	11	0	0,00	0	N/A
X	Caenorhabditis elegans (worm)	0.25	76%	13794	6	12	1	0.03	307	0,0307

Times found – how many times at least the same number of G4s as for actual genome was found among 10,000 generated genomes

One tailed p-value – measured p-value (times found/10,000)

SUPPLEMENTARY REFERENCES

1. Brzezniak, L. K., Bijata, M., Szczesny, R. J. & Stepien, P. P. Involvement of human ELAC2 gene product in 3’ end processing of mitochondrial tRNAs. *RNA Biol.* **8,** 616–626 (2011).
2. Bedrat, A., Lacroix, L. & Mergny, J.-L. Re-evaluation of G-quadruplex propensity with G4Hunter. *Nucleic Acids Res.* **44,** 1746–1759 (2016).
3. Kwok, C. K. & Balasubramanian, S. Targeted Detection of G-Quadruplexes in Cellular RNAs. *Angew. Chem. Int. Ed Engl.* **54,** 6751–6754 (2015).
4. Rachwal, P. A. & Fox, K. R. Quadruplex melting. *Methods San Diego Calif* **43,** 291–301 (2007).
5. Nieradka, A. *et al.* Grsf1-induced translation of the SNARE protein Use1 is required for expansion of the erythroid compartment. *PloS One* **9,** e104631 (2014).
6. Szczesny, R. J. *et al.* Identification of a novel human mitochondrial endo-/exonuclease Ddk1/c20orf72 necessary for maintenance of proper 7S DNA levels. *Nucleic Acids Res.* **41,** 3144–3161 (2013).
7. Liu, Q., Greimann, J. C. & Lima, C. D. Reconstitution, activities, and structure of the eukaryotic RNA exosome. *Cell* **127,** 1223–1237 (2006).
8. Kikin, O., D’Antonio, L. & Bagga, P. S. QGRS Mapper: a web-based server for predicting G-quadruplexes in nucleotide sequences. *Nucleic Acids Res.* **34,** W676-682 (2006).
