# Supplementary material for: Dedicated surveillance mechanism controls G-quadruplex forming non-coding RNAs in human mitochondria
Source: Nat Commun. 2018 Jul 2;9:2558. doi: 10.1038/s41467-018-05007-9 (PMC6028389; doi:10.1038/s41467-018-05007-9)
Supplement: Supplementary file 3 — Description of Additional Supplementary Files [file 41467_2018_5007_MOESM3_ESM.docx]

**Description of Additional Supplementary Files**

File Name: Supplementary Data 1

Description: Mass spectrometry results of degradosome co-purification studies.
